# Integrating Transcriptomic and Metabolomic Analyses to Explore the Effect of Color Under Fruit Calyx on That of Fruit Apex in Eggplant (*Solanum melongena* L.)

**DOI:** 10.3389/fgene.2022.889461

**Published:** 2022-06-23

**Authors:** Jingjing Zhang, Bing Li, Xiurui Gao, Xiuqing Pan, Yanrong Wu

**Affiliations:** Institute of Cash Crops, Hebei Academy of Agriculture and Forestry Sciences, Shijiazhuang, China

**Keywords:** transcriptomic, metabolomic, eggplant, color, anthocyanin

## Abstract

Fruit color is an important commercial characteristic of eggplant (*Solanum melongena* L.), which affects both the profits of growers and consumer choice. Two eggplant inbred lines were discovered: “Z,” which is a light purple color under the fruit calyx, with purple on the fruit apex; and “L,” fruits of which are green under the calyx and at the apex. To determine the molecular mechanisms underlying the effect of fruit peel color under the calyx on that at the fruit apex, we conducted a combined transcriptomic and metabolomic analyses of the Z and L inbred eggplant lines. Transcriptome analysis of peel samples from three fruit regions (under the calyx, the apex, and the middle surface) of each line was conducted by RNA sequencing, and generated a total of 791,512,404 clean reads from 18 samples (three biological replicates). Differentially expressed genes (DEGs; n = 424) were identified in comparisons of peel samples from the three sites of L line fruits. Gene ontology analysis showed that “catalytic activity” was extremely significantly enriched. Further, DEGs (n = 8) were enriched in the Kyoto Encyclopedia of Genes and Genomes pathway “flavonoid biosynthesis.” Levels of *CHI*, *LDOX*, *F3′5′H*, and *dihydroflavonol reductase* were higher in the Z line than the L line. In addition, metabolome analysis showed that, 10 differentially accumulated metabolites were detected between peel samples from the apex of L and Z line fruit. The most significant DAM was delphinidin-3-O-rutinoside (Z line content, 34.89 μg/g vs*.* L line content 0.01 μg/g). Combined transcriptomic and metabolomic analyses indicated that *DFR* and *F3′5′H* were closely related to content of the metabolites, cyanidin and delphinidin, and that some downstream metabolites differed significantly between the L and Z lines. Content levels of delphinidin-3-O-rutinoside, delphinidin-3-O-glucoside, cyanidin-3-O-glucoside, and cyanidin-3-O-rutinoside were markedly down-regulated in the L line. Altogether, increased *CHI* levels could up-regulate the downstream genes, *LDOX, F3′5′H*, and *DFR*, which further lead to increasing the content of delphindin. Thus, the uniform purple color was presented at the apex of fruits in Z plants. These findings not only identify key candidate genes, but will also improve understanding of the genetics and the efficiency of breeding for eggplant fruit color.

## Introduction

Eggplant (*Solanum melongena* L*.*) is an important vegetable that is widely cultivated and consumed throughout the world (Cericola et al., 2014; Zhou, 2016). There are many different colors of eggplant, including white, green, blackish-purple, purplish-red, purple, and orange (Lv et al., 2019; Tian et al., 2019). Coloration is an important quality of eggplant, which has an important influence on consumption habits in different areas. For example, in China, people from the northeast region like long purple/red eggplant, while those from the north tend to prefer round purple-black eggplant; however, in weak light, the fruit apex of purple eggplant often appears non-uniform and green in color (Xiang et al., 2015), which seriously affects its commercial value. Most consumers prefer eggplant with uniform color of fruit top and fruit surface. We found that the fruit with uneven color of fruit apex and green color showed green under calyx, while the fruit with uniform color of fruit apex showed light purple under calyx. According to the discovery, we speculated that there is a certain connection between the color of fruit apex and fruit under calyx. Until now, the reason of this phenomenon and mechanism are unexplored. Therefore, study of the cause of eggplant fruit apex greening and breeding of high-quality eggplant varieties with uniform fruit color are highly desirable.

Most plant colors are primarily determined by flavonoids (Wang et al., 2021), which include chalcone, flavone, flavonol, isoflavone, flavanone, and anthocyanin, and are important secondary metabolites (Brenda, 2001), beneficial to both fruit color development (Zhang et al., 2020) and fruit quality (Ravaglia et al., 2013). Anthocyanins, including cyanidin, delphinidin, pelargonin, petunidin and so on, are among the flavonoids with major roles in fruit color, which support the development of purple, blue, and red colors (Jaakola, 2013). Anthocyanins are synthesized from phenylalanine, then the substrate, 4-coumaroyl-CoA, is catalyzed by chalcone synthase (CHS) and chalcone isomerase (CHI), which generate naringenin. Next, the enzymes flavanone 3-hydroxyl (*F3*H), leucoanthocyanidin dioxygenase (LDOX), flavonol synthase (FLS), and dihydroflavonol reductase (DFR), catalyze cyanidin, delphinidin, and pelargonidin production (Sakuta, 2014). Anthocyanin biosynthesis genes have been identified in many plant species, including *Salvia miltiorrhiza* Bge (Jiang et al., 2020), jujube (Shi et al., 2020), grape (Shi et al., 2020), turnips (Li et al., 2020), and eggplant (Li et al., 2018). Anthocyanin biosynthesis genes include those encoding structural factors, such as *F3H*, *CHS*, *CHI*, *DFR*, *ANS* (Li et al., 2020), and *UFGT* (Li et al., 2020), as well as transcription factors (TFs), and several related regulatory genes, including *MYB* (Cheng et al., 2021; Yan et al., 2021), *bHLH* (Xi et al., 2021), and *WD40* (Gao et al., 2021).

The content of anthocyanins in purple eggplant is higher than that in white and green eggplant (Tian et al., 2019), and 2.34 and 7.08 times higher than those in grapes and red onions, respectively (Wu et al., 2006). Recently, deeper research into eggplant anthocyanins has been reported, including functional characterization of eggplant TFs (Xi et al., 2021; He et al., 2021; Moglia et al., 2021) and expression of genes related to eggplant anthocyanin biosynthesis (Zhang et al., 2020). Further, cloning and expression analysis of eggplant biosynthesis-associated genes has been reported (Wang et al., 2017; Tian et al., 2019); however, the molecular underpinnings of eggplant fruit color under the calyx and at the apex has yet to be reported and warrants further study. In recent years, eggplant peels have attracted more and more attention for their anthocyanins. Because of that, more and more experiments have emerged to study anthocyanins in eggplant peels. For instance, comparing six different colors of eggplant peels was conducted by Yang et al. (2022). The gene of *F3′5′H* expression level was higher in dark-purple eggplant peels than in others, which was consistent with delphinidin content in anthocyanins. Transcription factor *SmMYB113* promotes anthocyanin biosynthesis in eggplant (Yang et al., 2022). *SmTT8* interacts with transcription factor *SmMYB75* to promote anthocyanin accumulation (Shi et al., 2020). Transcription factor *SmbHLH1* represses anthocyanin accumulation in eggplant (Duan et al., 2021). Although many researchers have made in-depth studies on eggplant peel, the investigations on different parts of eggplant peel are still scarce.

In recent years, genomics databases have been constructed for numerous plants (Song et al., 2020; Wei et al., 2020), and transcriptome sequencing combined with metabolome analysis technology has been widely applied to research the biosynthesis of metabolites in such plants (Jiao et al., 2020; Zhou et al., 2020; Wang et al., 2021; Wu et al., 2021; Zhang et al., 2019; Zhuang et al., 2019). Transcriptome sequencing can be enriched by functional annotation from various databases, and metabolic pathways related to phenotypes can be deeply explored. Then, the metabolite content of target compounds in metabolic pathways can be determined and analyzed by targeted metabolome analysis technology.

Two eggplant inbred lines were discovered by the Institute of Cash Crops, Hebei Academy of Agriculture and Forestry Sciences: “Z,” which is a light purple color under the fruit calyx, with purple on the fruit apex; and “L,” fruits of which are green under the calyx and at the apex, they are the same color as soon as they begin to grow. In the present study, spontaneous mutations in the eggplant inbred line Z and its wild-type counterpart, line L, were investigated. Eggplant peel was assessed by transcriptome sequencing and metabolome analysis to identify metabolite biosynthesis pathways that could explain differences in coloration of the peel at the fruit apex between the two lines. This study both explains fruit color uniformity and provides a theoretical basis for eggplant fruit color breeding.

## Materials and Methods

### Plant Materials and Treatments

**FIGURE 1:**
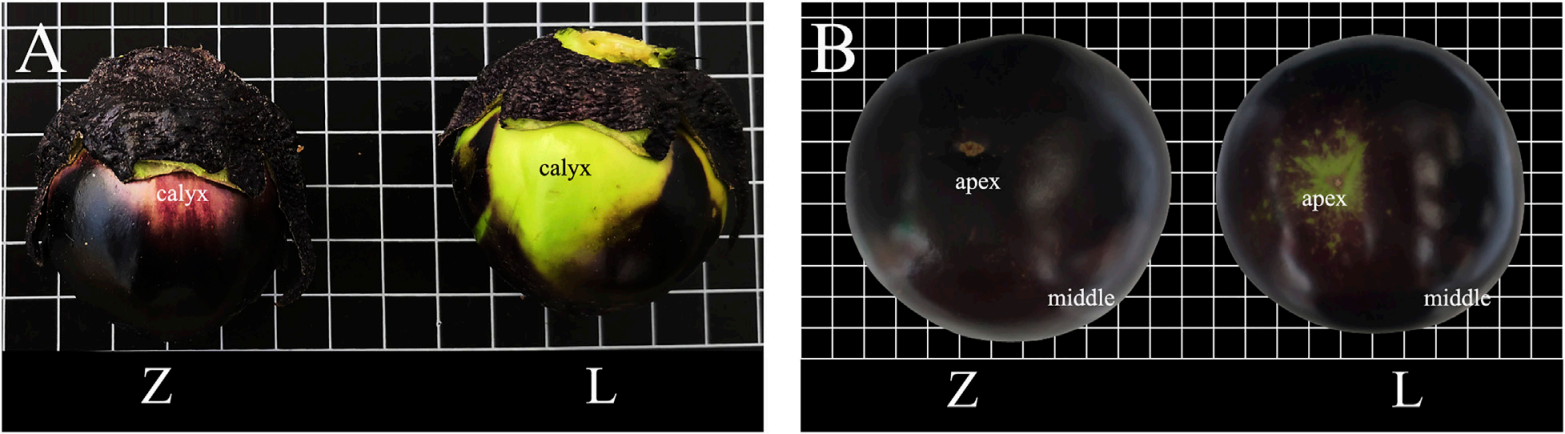
The two inbred eggplant (*Solanum melongena* L.) lines, Z and L. **(A)** Peel color under the calyx of Z and L fruits. **(B)** Peel color at the apex of Z and L fruits.

Two eggplant inbred lines, with light purple fruit color under calyx (Z) and green fruit color under calyx (L), bred by the Institute of Cash Crops, Hebei Academy of Agriculture and Forestry Sciences, were used as materials. Z was selected as a spontaneous mutation of the inbred line, L, and has a purple fruit apex and peel, while fruit apex is green and the peel is purple in Line L (Figures 1A,B). On 8 January 2018, seedlings of Z and L were sown in trays in a solar greenhouse in Shijiazhuang City (114.26E, 38.03 N), Hebei province, China. On 20 March 2018, seedlings were transplanted to a plastic tunnel. When fruit were 5–7 cm in diameter (6 June 2018), peel samples were collected from under the calyx (Le and Ze), the apex (Ld and Zd), and the middle surface (Lm and Zm) of eggplant lines L and Z, respectively (Supplementary Figure S1). Three biological replicates, each comprising peel samples from three fruits, were prepared, frozen by using liquid nitrogen, and then stored at −80 °C for transcriptome and metabolome analyses.

### RNA Extraction and Transcriptome Sequencing

Total RNA was extracted from each sample using a DP441 Kit (Tiangen, China), according to the manufacturer’s instructions. RNA quality was evaluated by 1% agarose gel electrophoresis, and RNA was purified using AMPure XP (IMPLEN, United States). RNA concentrations and RNA integrity were determined using a Qubit RNA Assay Kit and a Qubit 2.0 Fluorometer (Life Technologies, United States) and the RNA Nano 6000 Assay Kit with an Agilent Bioanalyzer 2100 (Agilent Technologies, United States), respectively. RNA was sequenced using the Illumina HiSeq high throughput sequencing platform (Illumina HiSeq 4000, United States). RNA extraction and transcriptome were completed by Beijing Novogene Bioinformatics Technology Co., Ltd., (Beijing, China).

### Transcriptome and Differential Gene Expression Analyses

Clean reads were obtained by removing those containing adapter sequences, N bases, and low quality reads among raw reads from the Illumina HiSeq 4000, as quality control. Clean reads were aligned to eggplant reference sequences (ftp://ftp.kazusa.or.jp/pub/eggplant/SME_r2.5.1.fa.gz; Hirakawa et al., 2014) using HISAT software (http://ccb.jhu.edu/software/hisat/index.shtml). Gene expression levels were analyzed using HTSeq (https://htseq.readthedocs.io/en/master/) using the FPKM method. Third, differential gene expression was analyzed using DESeq (Andrea et al., 2010), and differentially expressed genes (DEGs) screened according to fold-change and the level of significance of the difference level, with threshold selection criteria of | log_2_ (fold-change) | > 1 and q-value < 0.05. Gene Ontology (GO) enrichment of obtained DEGs was analyzed using the GOseq R package and Kyoto Encyclopedia of Genes and Genomes (KEGG) enrichment evaluated using KOBAS (2.0) (Mao et al., 2005).

### Real-Time Quantitative PCR (qRT-PCR)

Total RNA was extracted from peel samples using Trizol and detected by 1% agarose gel electrophoresis. Reverse transcription was performed using a CWbio RT Reagent kit, following the product instructions. Gene-specific primers were designed using Primer Premier V5.0 (Primer, Canada) (Supplementary Table S1). Then, qRT-PCR was conducted on an ABI 7500 Real-Time PCR system (Applied Biosystems, United States), using the following program: 95 °C for 15 min, followed by 40 cycles of 95 °C for 10 s and 72 °C for 30 s. Every reaction contained 10 μL 2× SuperReal PreMix Plus, 100 ng cDNA, 1.2 μM gene-specific primers, and RNase-Free ddH_2_O, in a final volume of 20 μL.

### Anthocyanin Extraction and Metabolome Detection

Fruit peel samples from under the calyx (Le and Ze) and the apex (Ld and Zd) of eggplant lines L and Z, respectively, were freeze-dried in a vacuum and crushed using a mixer mill MM400 (Retsch, Germany) containing zirconia beads at 30 Hz for 15 min. Then, 50 mg powder was dissolved in 80% methanol (0.1% HCl), vortexed for 10 min, and treated with ultrasound for 10 min. Tissue homogenates were then centrifuged at 12000 r/min (4 °C, 3 min) and supernatants collected and filtered through a 0.22 µm pore-size filter (Shanghai Anpu Experimental Technology Co., LTD.).

Anthocyanin extracts were analyzed using an ultra-performance liquid chromatography and tandem mass spectrometry (UPLC-MS) system at Wuhan Metware Bioinformatics Technology Co., Ltd (Wuhan, China). The UPLC column was a Waters ACQUITY BEHC18 (1.7 µm, 2.1 × 100 mm), solvent system A was water (0.1% formic acid) and system B was methanol (0.1% formic acid). The gradient program was as follows: solvent system B, 5% at 0 min, 50% at 6 min, 95% at 12 min (held for 2 min), and finally decreased to 5% at 14 min and held for 2 min. The temperature of the column was 40 °C, the injection volume of anthocyanin extracts was 2 μL, and the flow rate was 0.35 ml/min. The effluent was alternatively connected to an electrospray ionization (ESI)-triple quadrupole-linear ion trap (QTRAP)-MS. The temperature of the ESI was 550 °C, the ion spray voltage was 5500 V in positive ion mode, and curtain gas was 35 psi. In the Q-Trap 6500+, the declustering potential and collision energy for individual multiple reaction monitoring (MRM) transitions was optimized; a specific set of MRM transitions were monitored for each period, according to the metabolites eluted within the period.

### Anthocyanin Standard Curve Construction

Anthocyanin standards were prepared at various concentrations (0.01, 0.02, 0.05, 0.1, 0.5, 1, 5, 10, 50, 100, 500, 1,000, 2000, and 5,000 ng/ml) and mass spectrum peak intensity data for the corresponding quantitative signal of each concentration standard obtained. Standard curves of different anthocyanins were drawn according to the concentration and peak area of the standard substance.

### Anthocyanin Analyses

The content of substances in experimental samples was calculated based on standard curve equations (Supplementary Table S2). Unsupervised principal component analysis was performed using the statistics function, prcomp, in R (www.r-project.org). Hierarchical cluster analysis results from samples and metabolites are presented as heatmaps with dendrograms, with significantly regulated metabolites between groups defined by variable importance in the projection score ≥0 and absolute Log_2_ fold-change ≥ 1.0, and identified metabolites annotated using the KEGG Compound database (http://www.kegg.jp/kegg/compound/). Annotated metabolites were then mapped to the KEGG Pathway database (http://www.kegg.jp/kegg/pathway.html).

## Results

### Transcriptome Sequencing

To provide a comprehensive overview of how transcripts related to eggplant fruit color under the calyx effect that of peel at the apex, 18 cDNA libraries from were sequenced by RNA-Seq. A total of 805,350,080 raw reads were obtained, with 791,512,404 clean reads after removal of low-quality reads and adapter sequences. Q20 and Q30 values indicate probabilities of an error in base recognition of 1% and 0.1%, and both were >90% in our dataset. (Supplementary Table S3). Of total clean reads, > 90% were completely aligned with the eggplant reference genome, as were >87% of unique reads (Supplementary Table S4). These data demonstrate that the sequence quality was sufficient for our experimental requirements.

### DEGs Screening

**FIGURE 2:**
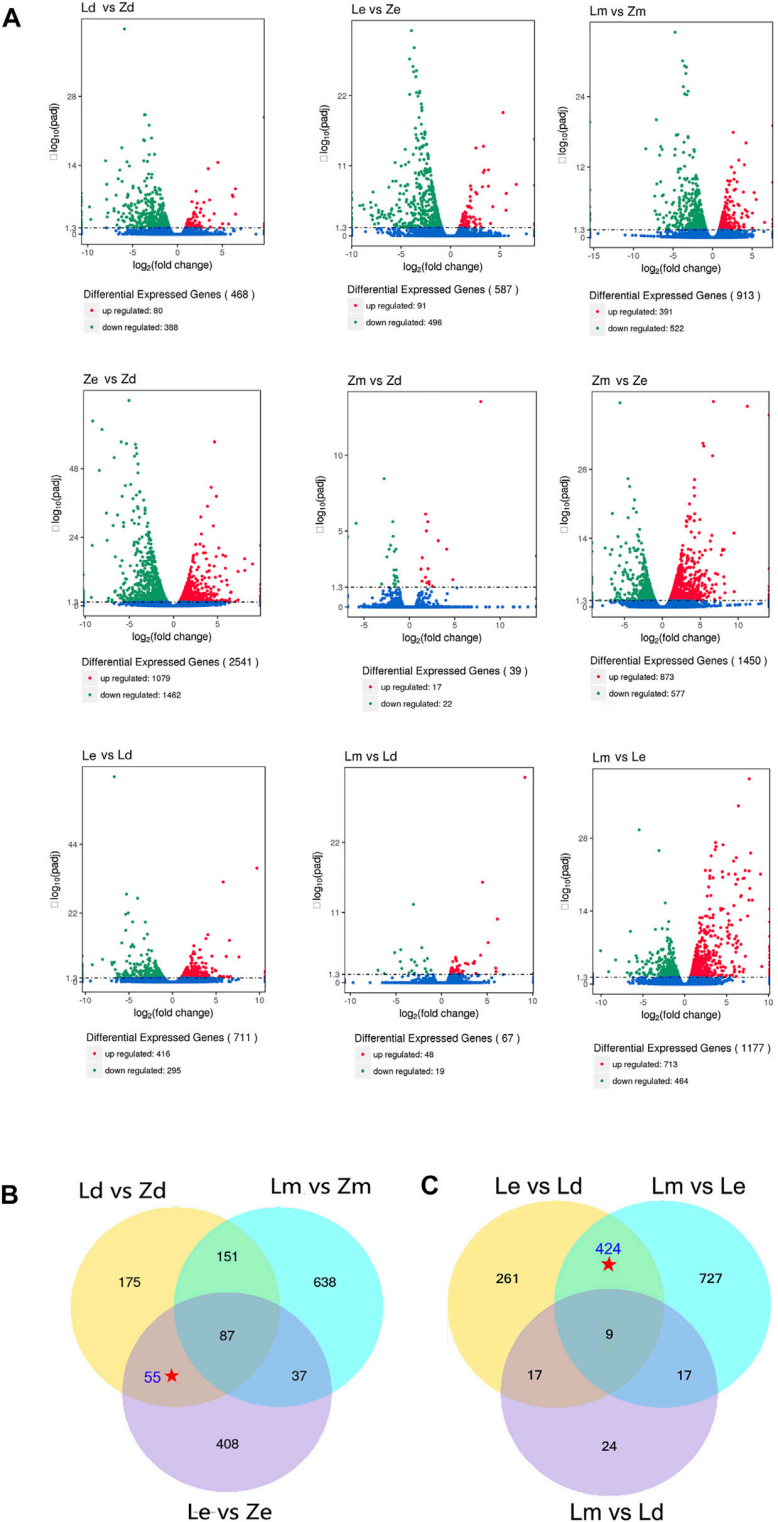
Analysis for differentially expressed genes (DEGs) among different combinations of eggplant peel samples collected from under the calyx (Le and Ze), the apex (Ld and Zd), and the middle surface (Lm and Zm) of eggplant fruit from lines L and Z, respectively. **(A)** The DEG numbers among different comparisons. **(B)** Venn diagram showing overlapping DEGs in comparisons of the same regions between the wild type and mutant. **Le**, peel under the calyx of L; **Ze**, peel under the calyx of Z; **Lm**, the peel on the middle parts of L; **Zm**, the peel on the middle parts of Z; **Ld**, the peel on apex parts of L; **Zd**, the peel on apex parts of Z. **(C)** Venn diagram showing overlapping DEGs in comparisons of peel samples from different parts of L line eggplant fruit. **Le**, peel under the calyx of L; **Ze**, peel under the calyx of Z; **Lm**, the peel on the middle parts of L; **Zm**, the peel on the middle parts of Z; **Ld**, the peel on apex parts of L; **Zd**, the peel on apex parts of Z.

DEGs were screened according to a threshold of q-value < 0.005 and | log_2_ (fold-change) | > 1.The highest number of DEGs was between samples from under the fruit calyx (Ze) and apex (Zd) in line Z (n = 2,541), while the number of DEGs between samples from under the fruit calyx and the fruit apex of L (Le vs*.* Ld) was 711. The lowest number of DEGs was between Zm and Zd (n = 39); there were 67 DEGs between Lm vs*.* Ld. There were 587 DEGs between samples from under the calyx of Z and L fruits, and 468 between the Z and L fruit apex samples (Figure 2A).

To compare the DEGs between the wild type (L) and mutant (Z), there were 587 DEGs in Le vs*.* Ze, 468 DEGs in Ld vs*.* Zd, and 913 DEGs in Lm vs*.* Zm. Comparing the Venn diagram (Figure 2B), there were 142 common DEGs between Le vs*.* Ze and Ld vs*.* Zd, while 87 common DEGs among Le vs*.* Ze and Ld vs*.* Zd and Lm vs*.* Zm. After excluding 87 the common DEGs, 55 DEGs remained which may influence the color of fruit apex and the color under the calyx.

To study the effect of the color of peel under the calyx on that of the fruit apex, DEGs between peel samples from different parts of L fruit were compared using a Venn diagram. There were 433 common DEGs between Le vs*.* Ld and Lm vs*.* Le. After excluding nine common DEGs between Lm vs*.* Ld, 424 DEGs remained which may influence the color of the fruit apex (Figure 2B).

### GO and KEGG Analyses of DEGs

**FIGURE 3:**
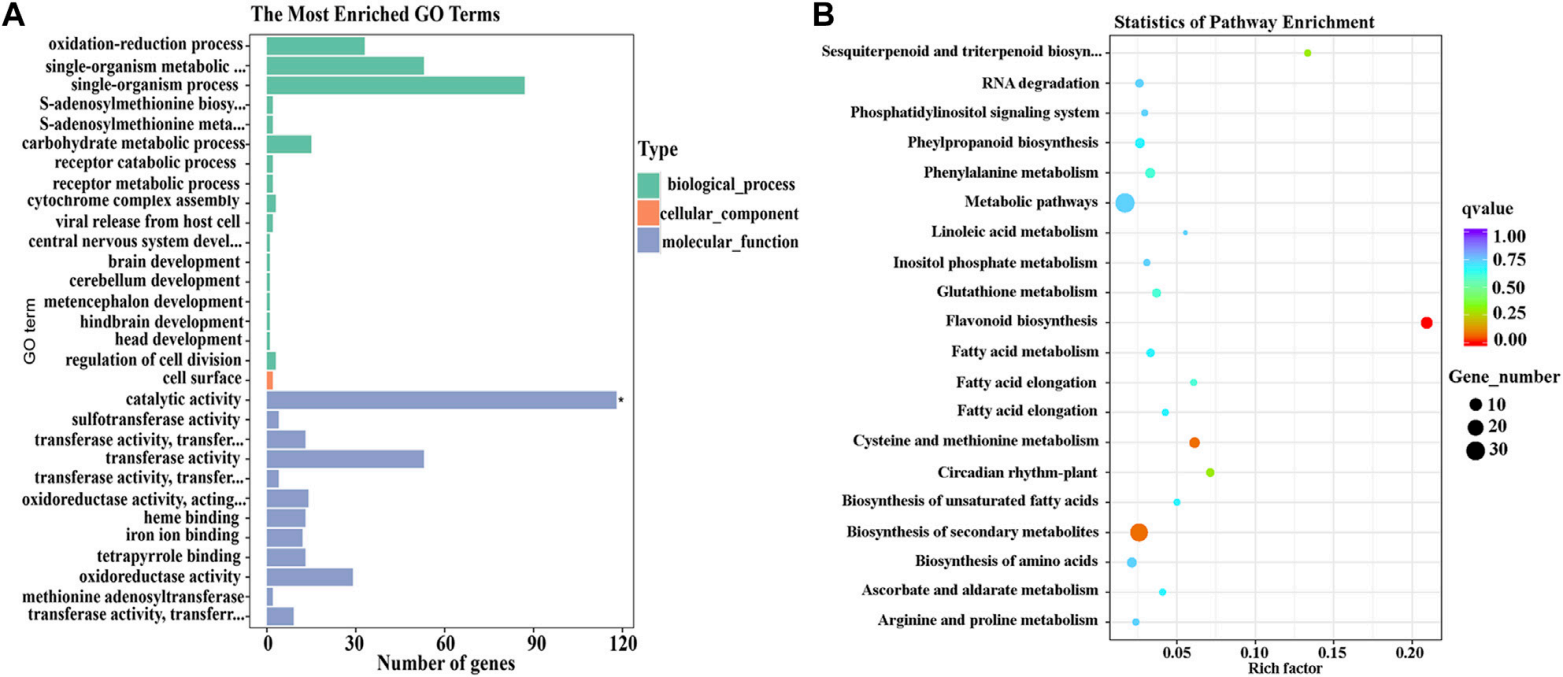
GO and KEGG pathways enriched for 424 differentially expressed genes (DEGs) that potentially influence eggplant fruit apex color. **(A)** Analysis of GO terms enriched for the 424 DEGs. **(B)** The top 20 KEGG pathways enriched for the 424 DEGs.

GO assignment was used to classify the functions of the 424 DEGs identified in different parts of L line eggplant peel. DEGs were annotated to biological, cellular, and molecular processes. Of the molecular function categories, ‘catalytic activity’ (GO:0003824) was extremely significantly enriched, including 118 DEGs (Figure 3A).

The 424 DEGs were also annotated using KEGG and 49 enriched metabolic pathways identified. The top 20 pathways with the most DEGs are listed in Figure 3B; the ‘flavonoid synthesis’ (SLY00941) pathway was significantly enriched (*p* < 0.05) (Supplementary Table S5).

### Analysis of Flavonoid Biosynthesis Pathway DEGs

Eight of the 424 DEGs identified as potentially influencing fruit apex color in line L were enriched in the flavonoid biosynthesis pathway, as follows: dihydroflavonol-4-reductase (*DFR*), 4,5,7-trihydroxyflavanone (*FL3H*), flavonoid 3′,5′-hydroxylase (*F3′5′H*), colorless anthocyanin dioxygenase (*LDOX*), flavonoid 3'mono-oxygenase (*F3PH*), flavonoid 3′,5'methyltransferase (*FAOMT*), flavonoid alcohol synthetase (*FLS*), and chalcone-dihydroflavonoid isomerase (*CHI*). Further, these eight DEGs exhibited consistent changes in eggplant lines L and Z; the *FL3H*, *F3′5′H*, *LDOX*, *FAOMT*, *DFR*, and *CHI* genes were up-regulated in all parts of Z relative to L, while *F3PH* and *FLS* were down-regulated. Among these eight DEGs, gene expression levels of *CHI*, *LDOX*, *FL3H*, *FAOMT, DFR*, and *F3′5′H* differed significantly between lines L and Z (Table 1).

**TABLE 1:** Expression analysis of genes related to flavonoid biosynthesis.

Gene	Gene ID	New gene ID	Le_fpkm	Ze_fpkm	Ld_fpkm	Zd_fpkm	Lm_fpkm	Zm_fpkm
*LDOX*	Sme2.5_01638.1_g00005.1	-	12.67	3197.92	481.29	5015.14	1475.30	7208.77
*LDOX*	Sme2.5_01638.1_g00003.1	-	0.82	21.26	5.66	39.90	9.42	47.68
*CHI*	Sme2.5_01193.1_g00009.1	Smechr1001862	10.75	1994.11	536.60	5854.16	2227.06	8725.88
*FL3H*	Sme2.5_00015.1_g00020.1	Smechr0202240	202.92	4398.82	1696.87	7137.10	3813.72	10457.96
*F3′5′H*	Sme2.5_04313.1_g00001.1	Smechr1201797	10.97	1656.89	477.05	3559.46	1510.36	4940.55
*FAOMT*	Sme2.5_00065.1_g00021.1	Smechr0902232	3.17	1325.40	205.17	2229.35	797.38	3130.98
*FLS*	Sme2.5_01643.1_g00012.1	Smechr0401078	15.72	9.52	326.17	198.01	189.85	153.29
*F3PH*	Sme2.5_01772.1_g00002.1	Smechr0302994	45.58	21.00	145.07	93.26	114.81	111.76
*DFR*	Sme2.5_01401.1_g00004.1	Smechr0202337	66.63	9966.89	1738.39	11523.90	4425.53	16885.32

“Gene ID,” data from the 2014 reference genome; “New Gene ID,” data from the 2019 reference genome.

### Transcription Factors

**FIGURE 4:**
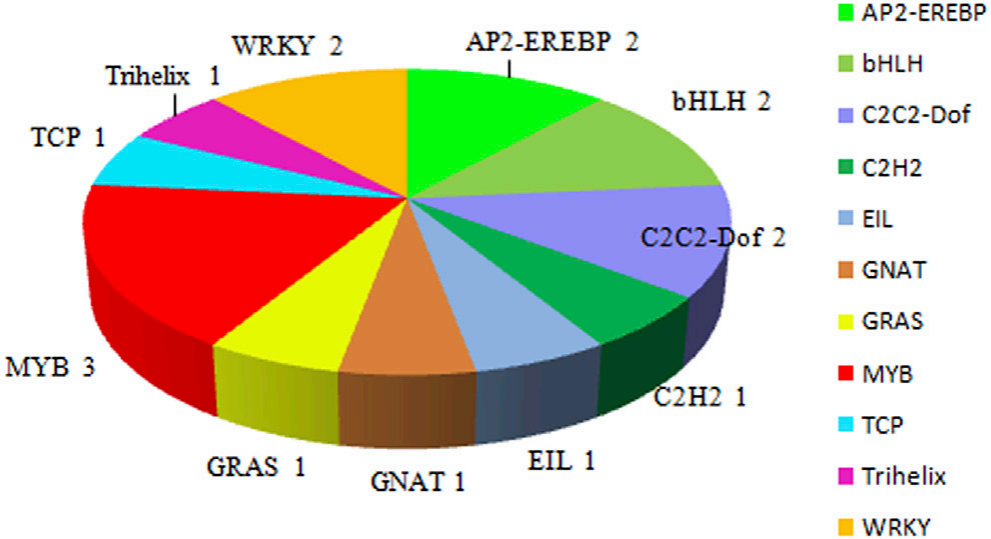
Transcription factor groups identified among the 424 differentially expressed genes.

Seventeen TFs were identified among the 424 DEGs, which were divided into nine TF families: MYB (three genes), AP2-EREBP (two genes), bHLH (two genes), C2C2-Dof (two genes), WRKY (two genes), C2H2 (one gene), EIL (one gene), GNAT (one gene), GRAS (one gene), TCP (one gene), and Trihelix (one gene) (Figure 4).

### qRT-PCR

**FIGURE 5:**
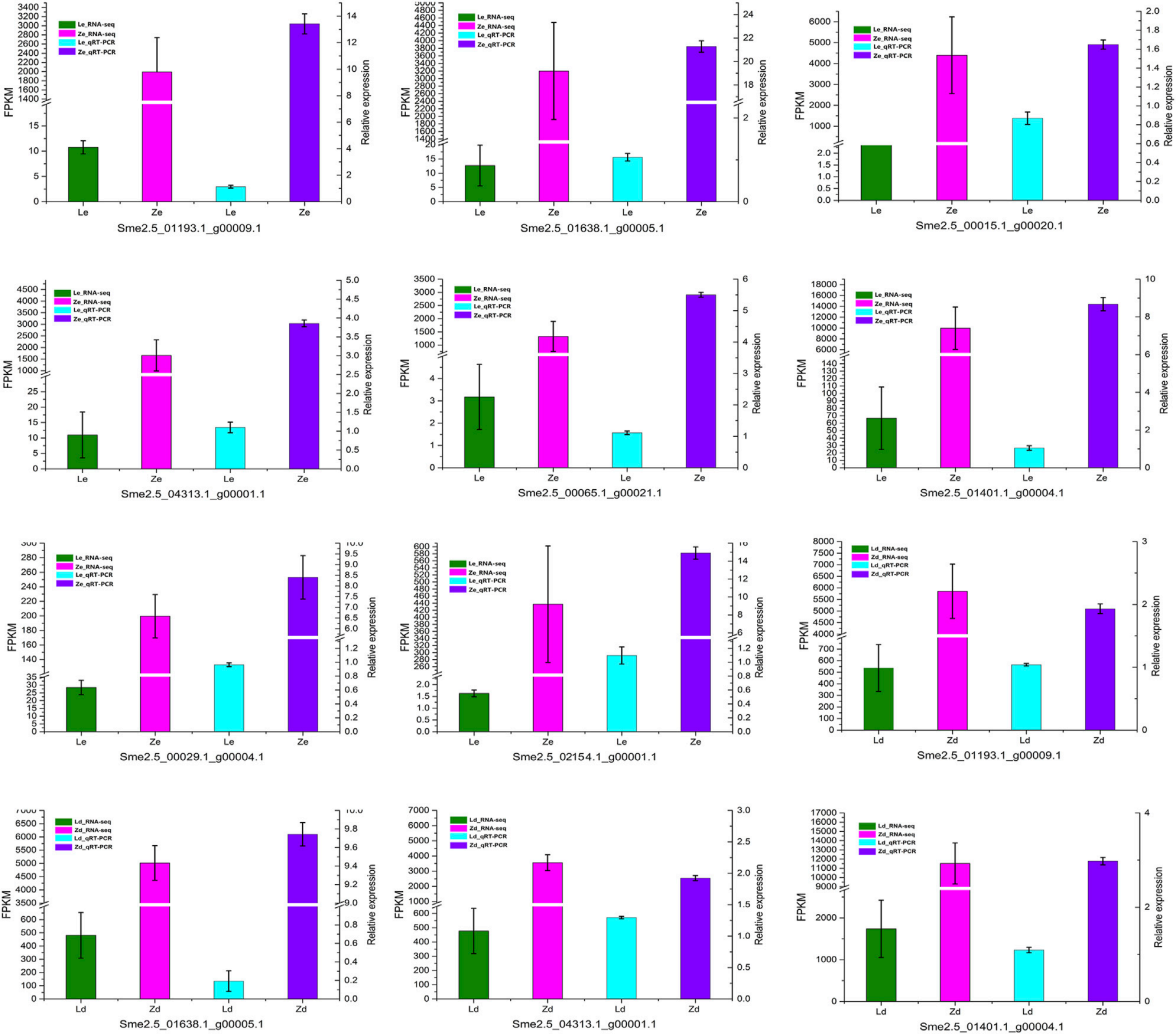
qRT-PCR confirmation of the differentially expressed genes identified by transcriptome analysis of peel samples from the fruit apex (Ld and Zd) and under the calyx (Le and Ze) of L and Z line eggplants, respectively.

To verify the reliability of transcriptomic sequencing data, the DEGs related to flavonoid metabolism were selected for qRT-PCR analysis; the results were consistent with the RNA-seq data (Figure 5).

### Differentially Accumulated Metabolites in Fruit Peel from Eggplant Lines L and Z

**FIGURE 6:**
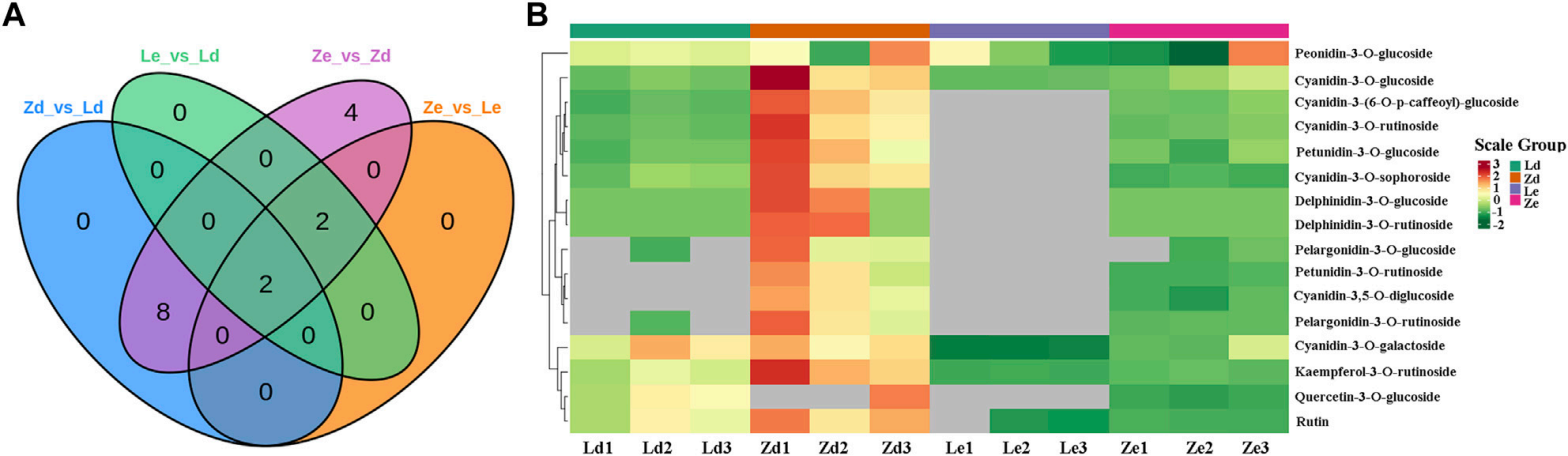
Analysis of differentially accumulated metabolites (DAMs) in eggplant peel samples from the fruit apex (Ld and Zd) and under the calyx (Le and Ze) of L and Z line eggplants, respectively. **(A)** Venn diagram showing overlapping DAMs in various comparisons of samples from L and Z lines. **(B)** Heatmap of DAMs involved in biosynthesis of anthocyanins.

Anthocyanin metabolites in fruit peel samples from the L and Z eggplant lines were detected by UPLC-MS. A total of 24 anthocyanins, including six cyanidins, five delphinidins, four flavonoids, two pelargonidins, three peonidins, and four petunidins, were detected in the samples. Further 10, 4, 16, and 4 differentially accumulated metabolites (DAMs) were detected between the Ld vs*.* Zd, Le vs*.* Ze, Ze vs*.* Zd, and Le vs*.* Ld groups, respectively (Figure 6A), associated with delphinidin, cyanidin, petunidin, and pelargonidin biosynthesis (Figure 6B).

**FIGURE 7:**
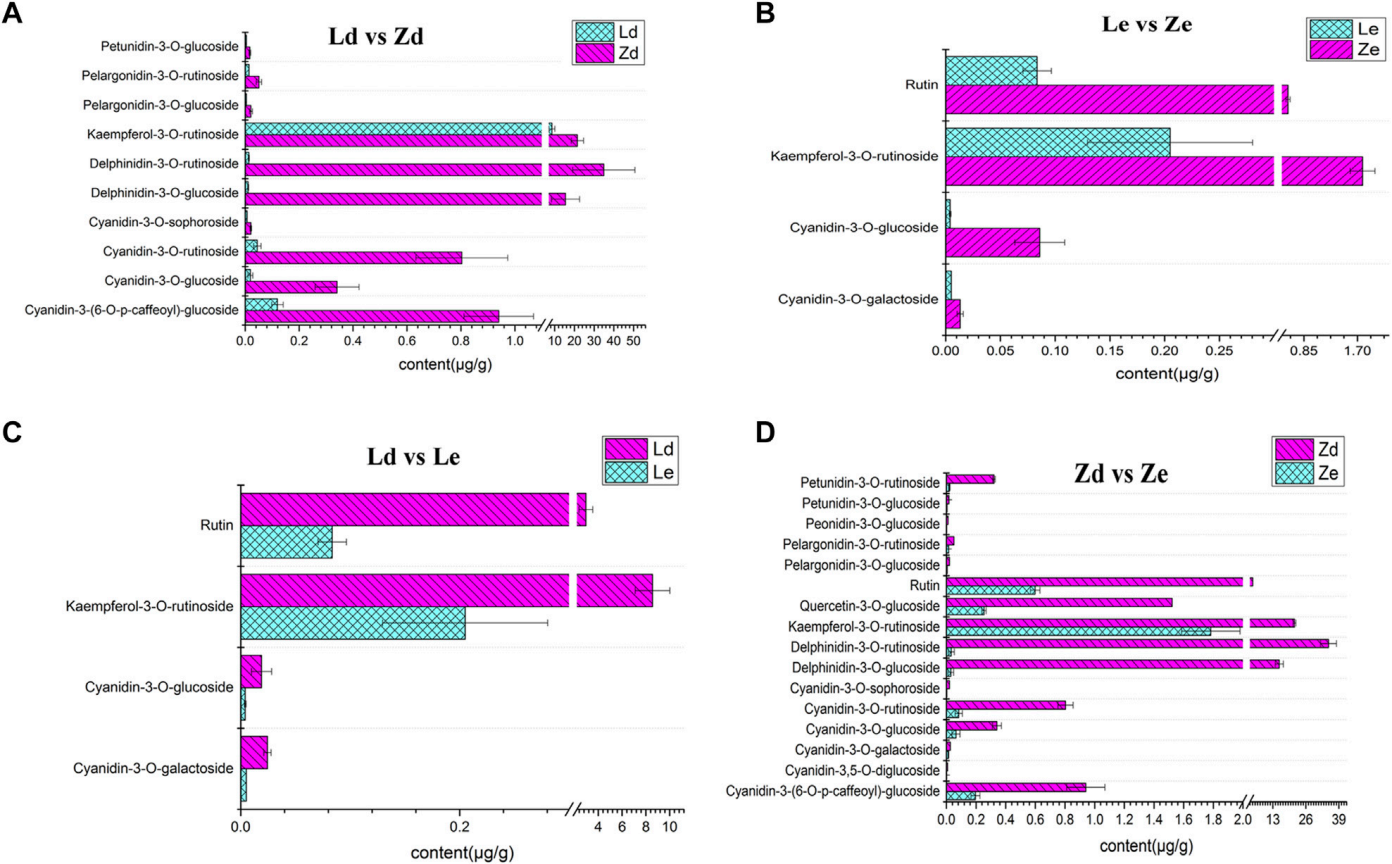
Content of various flavonoids and anthocyanins in eggplant peel samples from the fruit apex (Ld and Zd) and under the calyx (Le and Ze) of L and Z line eggplants, respectively. **(A)** Content of different metabolites in fruit peel from the apex of L (Ld) and Z (Zd) eggplants. **(B)** Content of different metabolites in fruit peel from under the calyx of L (Le) and Z (Ze) eggplants.**(C)** Content of different metabolites involved in fruit peel color between the apex (Ld) and under the calyx (Le) of fruits from L. **(D)** Comparison of the content of different metabolites involved in fruit peel color between the apex (Zd) and under the calyx (Ze) of fruits from Z.

Ten flavonoid and anthocyanin DAMs were detected between Ld and Zd fruit peels, as follows: kaempferol-3-O-rutinoside, cyanidin-3-(6-O-p-caffeoyl)-glucoside, cyanidin-3-O-glucoside, cyanidin-3-O-rutinoside, cyanidin-3-O-sophoroside, delphinidin-3-O-glucoside, delphinidin-3-O-rutinoside, pelargonidin-3-O-glucoside, pelargonidin-3-O-rutinoside, and petunidin-3-O-glucoside. The highest difference was in delthinidin-3-O-ruitinoside, the content of which was 34.89 μg/g in Zd, which was 3489-fold higher than that in Ld. The lowest difference was that in kaempferol-3-O-rutinoside content, which was 2.51-fold higher in Zd (21.50 μg/g) than in Ld (8.56 μg/g) (Figure 7A). There were four DAMs between Le and Ld: kaempferol-3-O-rutinoside, rutin, cyanidin-3-O-galactoside, and cyanidin-3-O-glucoside. Kaempferol-3-O-rutinoside content was 41.77-fold higher in Ld (8.56 μg/g) than in Le (Figure 7C). Further there were 16 flavonoid and anthocyanin DAMs between Ze and Zd, including: pelargonidin-3-O-glucoside, delphinidin-3-O-rutinoside, kaempferol-3-O-rutinoside, delphinidin-3-O-glucoside, and petunidin-3-O-rutinoside, among others. The DAM with the largest fold-change (671.50-fold) between Ze and Zd was pelargonidin-3-O-rutinoside. The metabolite with the highest content in Zd (34.89 μg/g) was delphinidin-3-O-rutinoside (Figure 7D). Cyanidin-3-O-galactoside, cyanidin-3-O-glucoside, kaempferol-3-O-rutinoside, and rutin content differed significantly between Ze and Le; for example, kaempferol-3-O-rutinoside content was 1.78 μg/g in Ze and 0.21 μg/g in Le (Figure 7B).

### Integrated Flavonoid and Anthocyanin Biosynthesis Transcriptome and Metabolome Analyses

**FIGURE 8:**
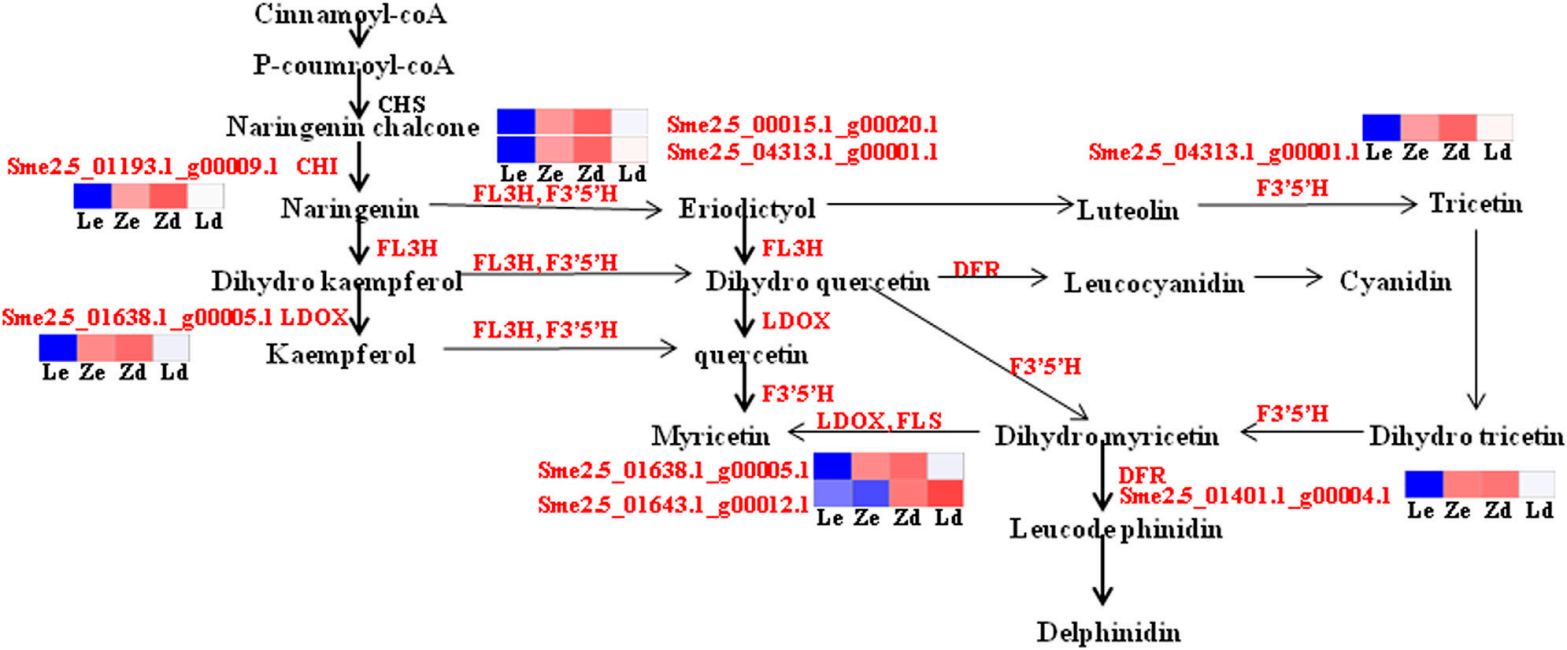
Detailed analysis of flavonoid biosynthesis pathways involved in eggplant fruit skin color based on transcriptome data. Ld and Zd, samples from the fruit apex, and Le and Ze, samples from under the calyx of L and Z line eggplants, respectively. Blue means down-regulated, red means up-regulated.

Six of the identified DEGs, including the upstream genes, *CHI* and *FL3H*, and the downstream genes, *LDOX*, *DFR*, *F3′5′H*, and *FLS*, are involved in flavonoid and anthocyanin metabolic pathways. *FLS* was down-regulated in the mutant Z line, in which the content of anthocyanins was higher than that in the wild-type L line, while the other genes were up-regulated. In the anthocyanin metabolism pathway, dihydroflavonol-4-reductase (*DFR*) and flavonoid 3′,5′-hydroxylase (*F3′5′H*) have important roles in the generation of terminal metabolites, such as delphinidins and cyanidins, which is regulated by the *DFR* and *F3′5′H* gene products (Figure 8).

**FIGURE 9:**
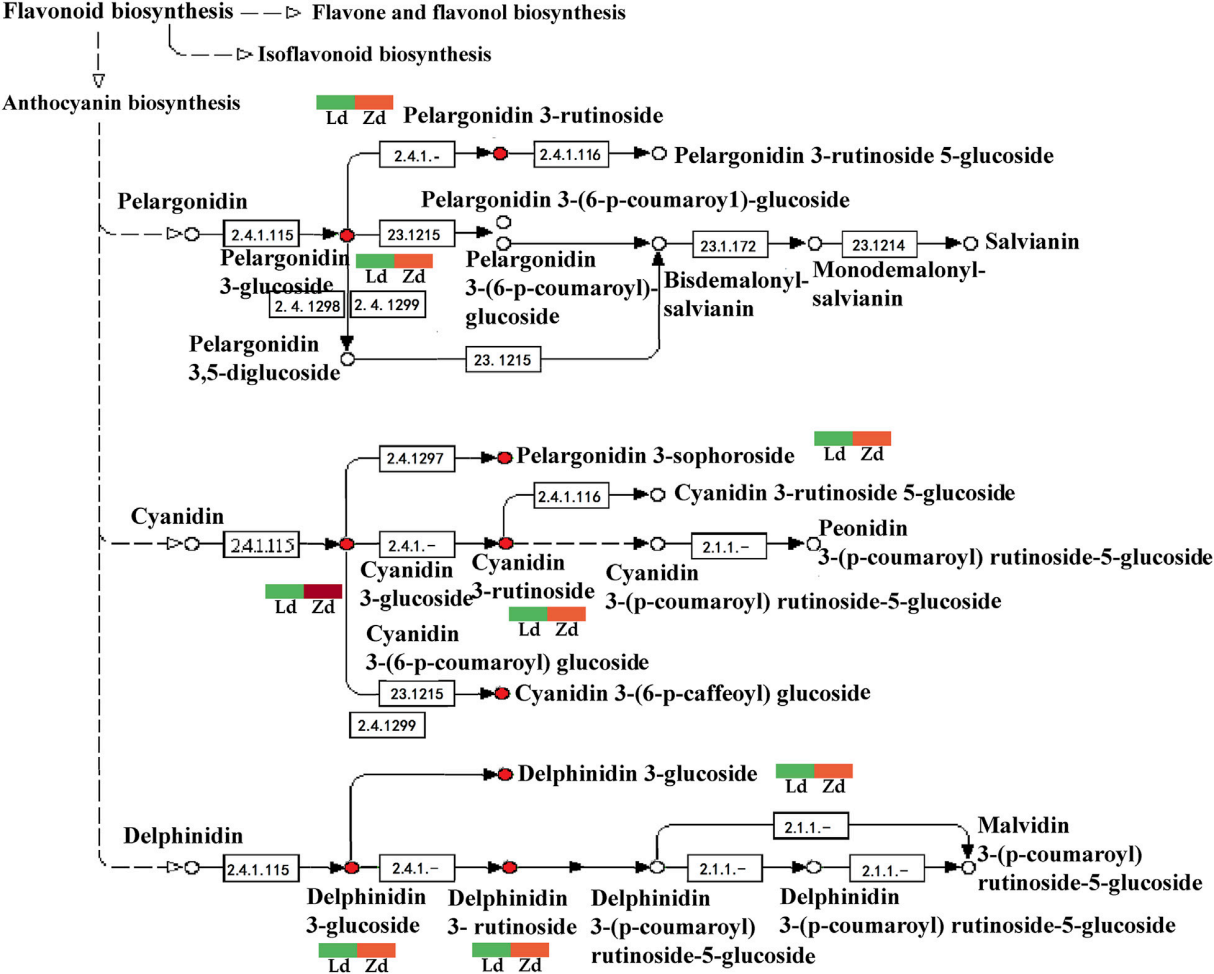
Detailed analysis of anthocyanin biosynthesis pathways based on metabolome data from peel samples from the apex (Ld and Zd) of L and Z line eggplant fruits, respectively. Green means down-regulated, red means up-regulated.

Delphinidin-3-O-glucoside, delphinidin-3-O-rutinoside, cyanidin-3-(6-O-p-caffeoyl)-glucoside, cyanidin-3-O-glucoside, cyanidin-3-O-rutinoside, cyanidin-3-O-sophoroside, kaempferol-3-O-rutinoside, pelargonidin-3-O-glucoside, pelargonidin-3-O-rutinoside, and petunidin-3-O-glucoside are involved in flavonoid biosynthesis, particularly anthocyanin pathways, contributing to fruit apex peel color in lines L and Z. Anthocyanin metabolites, including delphinidin-3-O-rutinoside and delphinidin-3-O-glucoside, were upregulated in the mutant line, Z (Figure 9).

As transcriptome analysis identified DEGs associated with flavonoid pathways, and particularly the anthocyanin pathway, flavonoids and anthocyanins were analyzed in eggplant peel. Content of the flavonoid, kaempferol-3-O-rutinoside, was higher in Zd than Ld, and is regulated by the early gene, *CHI*. Levels of the anthocyanins, delphinidin-3-O-glucoside, delphinidin-3-O-rutinoside, cyanidin-3-(6-O-p-caffeoyl)-glucoside, cyanidin-3-O-glucoside, cyanidin-3-O-rutinoside, and cyanidin-3-O-sophoroside, were higher in Zd than Ld, and are catalyzed by products of the later expressed genes, *F3′5′H* and *DFR*; these genes, as well as *CHI* and *LDOX*, were up-regulated in Zd vs*.* Ld.

## Discussion

Fruit color is an important trait, which is a focus of research attention because of its influence on the economic value of crops, and results from gene expression and metabolic content. Mutants are important for studying gene function (Singh et al., 2013). In the current study, we conducted integrated transcriptome and metabolome analyses of samples from Z and L line fruits, transcriptome data of peel samples from three fruit regions were analyzed to identify the key DEGs and metabolic pathways, and then the content of metabolites of peel samples under the calyx and at the apex was analyzed by metabolome, the results of which facilitate understanding of the effect of the color under eggplant fruit calyx on that of the fruit apex.

Chalcone isomerase (*CHI*), leucoanthocyanidin dioxygenase (*LDOX*), dihydroflavonol-4-reductase (*DFR*), and flavonoid 3′,5′-hydroxylase (*F3′5′H*) are key genes that determine flavonoid and anthocyanin metabolite content (Nan et al., 2021; Han et al., 2021; Li et al., 2021), which play important roles in color formation (Wang et al., 2018). *CHI* catalyzes the conversion of chalcones to flavanones (Chao et al., 2021; Ni et al., 2020) and the flavonoids generated include flavones, isoflavones, flavonols, and anthocyanidins (Nabavi et al., 2020). *CHI* may be a rate-limiting enzyme in this process, as its levels are correlated with anthocyanin content (Silvia et al., 2021). *LDOX* is the terminal enzyme of the anthocyanin biosynthesis pathway, which is mainly catalyzed by 2-ketoglutarate and Fe^2+^, transitioning from colorless to colored anthocyanidins (Appelhagen et al., 2011). Further, anthocyanin synthesis is promoted by increased *LDOX* gene expression (Wu et al., 2020). *DFR* and *F3′5′H* have important roles in anthocyanin regulation (Zhang et al., 2017; Wei et al., 2019; Li et al., 2021). In this study, compared with the peel under the calyx of L, levels of the *CHI*, *LDOX*, *DFR,* and *F3′5′H* genes were 185.46-, 252.34-, 149.59-, and 150.98-fold higher, respectively, in Z, and the trend in the fruit apex was consistent with that in samples from under the calyx at 10.91-, 10.42-, 6.23-, and 7.46-fold higher in Z. The expression levels of these genes on fruit surface showed the same trend as at fruit apex and under fruit calyx. In addition, the expression levels of *CHI*, *LDOX*, *DFR*, and *F3′5′H* genes in fruit surfaces were higher than those in fruit apex and under fruit calyx in both Z and L, and the expression levels of these genes in fruit apex were higher than those under the fruit calyx. These genes may have a cooperative relationship, and jointly regulate the development of eggplant peel color by producing different levels of secondary metabolites.

Besides, more and more transcription factors (TFs) were reported, and *MYB* transcription factors as the key transcriptional regulators played important roles in the regulation of anthocyanin biosynthesis (Yan et al., 2021). For example, *CaMYB306* accelerated fruit coloration in pepper (Ma et al., 2022). While *SmMYB86* and *StMYB44* palyed negative regulators of the anthocyanin biosynthesis in eggplant peels and potato tubers, respectively (Liu et al., 2019; Li et al., 2021). Additionally, *bHLH* TFs were also reported in regulation of anthocyanin biosynthesis (Duan et al., 2021; Zhang et al., 2021). In our study, *bHLH, C2C2-Dof, EIL*, and *GRAS* transcription factors were identified as significantly different expression levels. For example, the *bHLH* transcription factor was up-regulated both fruit apex and fruit under the calyx in Z. The expression level of which was 1,650.28 at the apex in Z while 241.97 in L, and 64.29-fold higher, respectively, under the calyx in Z. While the result was not consistent with the result of Duan et al. (2021), which might caused by the different region and period of sampling.

Anthocyanidins is one of the most important flavonoids (Nabavi et al., 2020), which include delphinidin, cyanidin, pelargonidin, petunidin, malvidin, and peonidin of six common types. Moreover, the purple or blue-red color is caused by delphinidin and the reddish-purple color is led by cyaniding in plants (Castañeda-Ovando et al., 2008). In previous research, the delphinidins were reported as the main anthocyanins in purple plants’ peels (Liang et al., 2022; Wang et al., 2021). In purple eggplant peels, delphinidin 3-O-glucoside (Yang et al., 2022), delphinidin-3-rutinoside (D3R) (Florio et al., 2021), delphinidin-3-(p-coumaroylrutinoside)-5-glucoside (Azuma et al., 2008) and delphinidin 3-O-rutinoside (Condurache et al., 2021) were enriched. They all belonged to the delphinidin though their structure were different. In our study, the most significant DAM was delphinidin-3-O-rutinoside at the apex of the fruits, (Z line content, 34.89 μg/g vs*.* L line content, 0.01 μg/g), which may be the key factor that caused the color distinction of the fruit apex.

In this study, by integrating transcriptome and metabolome, we found that kaempferol content increased with up-regulation of *CHI* and *LDOX* in Z, with delphinidins and cyanidins, particularly the metabolites delphinidin-3-O-glucoside, delphinidin-3-O-rutinoside, cyanidin-3-(6-O-p-caffeoyl)-glucoside, cyanidin-3-O-glucoside, cyanidin-3-O-rutinoside, and cyanidin-3-O-sophoroside, accumulating with increased *DFR* and *F3′5′H* expression, especially in eggplant Z fruit apex. During flavonoid biosynthesis, chalcone is converted to naringin via *CHI* activity, then naringin is converted to eriodictyol in response to up-regulation of the *FL3H* and *F3′5′H* genes, eriodictyol is converted to cyanidin when the *DFR* gene is upregulated, and to delphinidin in response to upregulation of *F3′5′H*. We speculate that mutations of *CHI*, *LDOX*, *F3′5′H*, and *DFR* may be a major reason underlying the different colors of lines L and Z. In addition, the *bHLH* transcription factor was up-regulated both fruit apex and fruit under the calyx in Z. Importantly, when the fruit is small, it is difficult to distinguish whether the fruit apex color is consistent with the fruit surface color if the material is selected through the fruit surface color, while if purple or light purple material is selected through the fruit top or under the calyx color, the fruit top color is consistent with the fruit surface color.

## Conclusion

In summary, transcriptome and metabolome were combined to reveal the relationship between color under eggplant fruit calyx and the color of the fruit apex in our study. We found that the genes *CHI*, *LDOX*, *F3′5′H*, and *DFR* are involved in eggplant fruit skin color formation. The differences in coloration between samples from the spontaneous mutant, Z, and the inbred line, L, may result from up-regulation of the *CHI*, *LDOX, F3′5′H*, and *DFR* genes in Z tissues. Increased *CHI* levels could upregulate the downstream genes, *LDOX, F3′5′H*, and *DFR*, leading to the accumulation of delphinidins, especially delphinidin-3-O-rutinoside and delphinidin-3-O-glucoside, which contributed to the purple color in the apex of fruits from Z plants. Altogether, this study provides a theoretical basis to explain variation in eggplant fruit color, and will promote the genetic breeding and manipulation of this commercially important trait.

## Data Availability

The original contributions presented in the study are publicly available. This data can be found here:PRJNA822006.
