# Supplementary figures and images for: Integrating Transcriptomic and Metabolomic Analyses to Explore the Effect of Color Under Fruit Calyx on That of Fruit Apex in Eggplant (Solanum melongena L.)
Source: Front Genet. 2022 Jun 23;13:889461. doi: 10.3389/fgene.2022.889461 (PMC9259842; doi:10.3389/fgene.2022.889461)

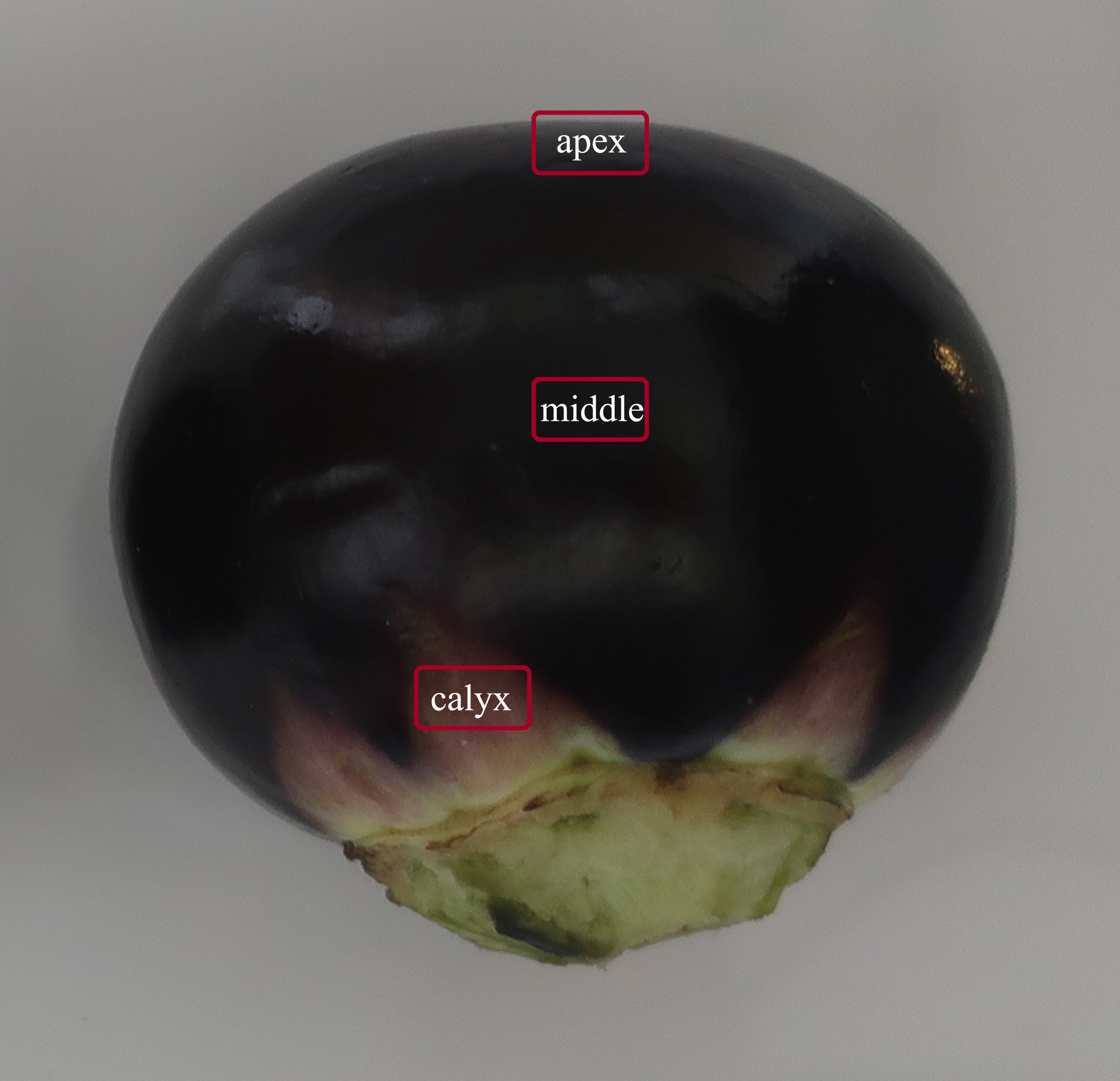

Supplement: Supplementary file 2 [file Image1.TIF]
